# Leveraging sensor technologies for seed phenotyping by genebanks

**DOI:** 10.3389/fpls.2025.1729121

**Published:** 2026-01-13

**Authors:** Kioumars Ghamkhar, David Rousseau

**Affiliations:** 1 New Zealand (NZ) Bioeconomy Science Institute - AgResearch Group, Grasslands Research Centre, Palmerston North,, New Zealand; 2 Université d’Angers, Laboratoire Angevin de Recherche en Ingénierie des Systèmes (LARIS), Angers, France

**Keywords:** climate change, genebanks, genetic diversity, high-throughput phenomics, seed phenotyping, sensor technologies, seed viability, digital agriculture

## Abstract

Genebanks serve as critical repositories for preserving the genetic diversity of plant species, including crops, forages, and their wild relatives, which is essential for adapting to climate change, enhancing food security, and improving agricultural sustainability. Seed phenotyping, the process of evaluating observable seed traits influenced by genetics and environmental factors, plays a pivotal role in characterizing and utilizing this diversity. Traditional phenotyping methods, however, are labor-intensive and inadequate for the vast collections housed in genebanks. This paper explores the transformative potential of high-throughput phenomics technologies, leveraging the electromagnetic spectrum—from gamma rays to radio waves—to enable rapid, precise, and non-invasive assessment of seed traits such as size, shape, biochemical composition, and vigor. We highlight the integration of advanced imaging systems (e.g., hyperspectral, X-ray, and thermal imaging) to enrich genebank datasets, facilitating trait discovery and crop improvement. Despite challenges like cost, scalability, and data standardization, opportunities arise from collaborative initiatives between genebanks and phenomics facilities through organizations such as International Plant Phenotyping Network. Our conclusions underscore how phenomics can revolutionize genebank operations, ensuring the efficient conservation and deployment of genetic resources to address global agricultural demands.

## 1 Introduction

Genebanks act as repositories for the genetic material of various plant species, including crops, forages, and their wild relatives. This conservation is crucial for maintaining the genetic diversity necessary for the adaptation and evolution of plant species. Genetic diversity within plant populations is vital for resilience against diseases, pests, and changing environmental conditions. Seed phenotyping and genebanks are pivotal components in the field of agriculture and plant science. Correct and reliable seed phenotyping at genebanks plays a crucial role in conserving plant genetic diversity, improving crop varieties, and ensuring food security (Bhattacharya and Mummenhoff, 2024; Ghamkhar et al., 2024).

Seed phenotyping is the process of assessing the observable characteristics of seeds influenced by their genetic makeup and environmental factors. It enables researchers and breeders to identify and select traits beneficial for crop improvement. Traits such as drought tolerance, disease resistance, and enhanced nutritional content are essential for developing improved crop varieties that meet specific agricultural needs. By utilizing the data obtained from seeds stored in genebanks, breeders can assess and estimate some traits that will translate to whole plants in order to enhance agricultural productivity and sustainability (Mascher et al., 2019; Nguyen and Norton, 2020).

The genetic traits preserved in genebanks are indispensable for developing crop varieties resilient to climate change, pests, and diseases. Rapid identification and incorporation of beneficial traits into crops are crucial for adapting to the unpredictable and adverse effects of climate change (Hoffmann and Sgrò, 2011; Chapman et al., 2012). However, the vast amounts of genetic material held in genebanks, often comprising thousands of accessions for a species, present a significant challenge for traditional seed phenotyping methods (Hay and Sershen, 2021). Should a genebank use these traditional phenotyping methods, they would need hours of labor to achieve their phenotyping goals. Fast and high-throughput phenotyping technologies enable the rapid assessment of large numbers of seeds, accelerating the process of identifying valuable traits.

The sheer volume of accessions in genebanks and the need for frequent vitality tests and seed quality assurance required, necessitates efficient and scalable seed phenotyping methods (Ouyang et al., 2024; Wonggasem et al., 2024). High-throughput phenotyping platforms can process thousands of samples simultaneously, providing comprehensive data on seed traits (Colmer et al., 2020; Liu et al., 2024). This capability is crucial for managing and exploiting the extensive genetic diversity stored in genebanks. Advanced phenotyping technologies, such as imaging systems, sensors, and automated data analysis, offer greater precision and accuracy compared to manual methods. These technologies can capture detailed phenotypic information, including seed size, shape, color, and health, with high reproducibility (Zhang et al., 2018; Zhang and Zhang, 2018). Such detail is essential for making informed decisions in breeding and conservation efforts.

The acceleration enabled by high-throughput phenotyping of seeds is crucial for developing new crop varieties that can meet the urgent demands of food production and environmental sustainability. Furthermore, fast phenotyping generates large datasets that can be integrated with genomic information to gain deeper insights into the genetic basis of seed and seedling traits (Colmer et al., 2020). This integration supports data-driven research and innovation in genebanks, leading to more effective strategies for crop improvement and conservation (Ghamkhar et al., 2024).

## 2 Traditional and current seed phenotyping methods

Traditional seed phenotyping methods encompass a range of manual, visual and/or destructive methods. Among these methods are the 100-seed weight measure, germination tests, tetrazolium staining, seed dimension measurement as well as other methods such as cut-tests and oven drying methods. There are also well established but non-traditional methods, which are early stages of non-destructive approaches.

### 2.1 100-seed weight

The 100-seed weight method is one of the most widely used traditional seed phenotyping approaches and provides a simple yet informative measure of seed size, density, and overall seed lot quality. In this method, a random sample of one hundred seeds is weighed, usually after equilibrating moisture content, and the resulting value is used as an indicator of seed development, uniformity, and potential vigor (Nautiyal, 2009). Because seed weight correlates with nutrient reserves and early seedling growth (Vaughton and Ramsey, 2001), it is often used in breeding programs and genebank characterization to compare accessions or detect deterioration in storage. Despite its usefulness, the method is labor-intensive, destructive at large scales, and limited in its ability to capture within-sample variability or internal defects.

### 2.2 Germination tests

The germination test is a cornerstone of traditional seed phenotyping and remains the most widely used method for assessing seed viability and vigor. In this test, seeds are placed under controlled temperature, moisture, and light conditions, and the proportion that successfully produce normal seedlings within a defined period is recorded. Because it directly measures a seed’s ability to initiate growth, the germination test is considered a biological gold standard for viability assessment in both genebanks and seed testing laboratories. However, it is time-consuming, often requiring days to weeks, labor-intensive, and inherently destructive, making it inefficient for large genebank collections (Hay and Whitehouse, 2017). Moreover, germination outcomes can be influenced by dormancy, subtle pathogen loads, or suboptimal test conditions, complicating interpretation (Finch-Savage and Leubner-Metzger, 2006).

### 2.3 Tetrazolium staining

The tetrazolium (TZ) staining test is a long-established biochemical method used to assess seed viability by detecting the metabolic activity of living tissues (Verma and Majee, 2013). In this procedure, seeds are cut or pierced to expose the embryo and then soaked in a tetrazolium chloride solution, where viable cells reduce the colorless compound to a red formazan pigment. The resulting staining pattern enables trained analysts to distinguish living, damaged, and non-viable tissues, making the method especially valuable for species with deep dormancy or slow germination, where standard germination tests may underestimate viability (França-Neto and Krzyzanowski, 2022).

### 2.4 Seed dimension measurement

Seed dimension measurement, typically involving manual assessment of seed length, width, and thickness using calipers or simple imaging tools, has long been a fundamental component of traditional seed phenotyping. These measurements provide basic morphological descriptors that are useful for taxonomic identification, detecting varietal purity, monitoring seed lot uniformity, and understanding traits related to seed dispersal or early seedling performance. However, manual dimension measurement is slow, labor-intensive, and often limited to a small number of seeds per batch, which reduces its ability to capture within-lot variability. It also provides only coarse information about external shape and cannot detect internal defects or subtle morphological differences.

### 2.5 Cut-tests and oven drying

Traditional seed quality assessments also often rely on cut-tests, oven-drying, and manual X-ray evaluation, each providing important but limited insights into seed integrity and composition. Cut-tests involve physically slicing seeds to visually inspect the embryo and endosperm, allowing rapid identification of empty, damaged, or insect-infested seeds, but at the cost of destroying the sample (Bhattacharya and Mummenhoff, 2024). Oven-drying methods, used to determine seed moisture content or to validate fill percentage, require controlled drying conditions and multiple handling steps, making them slow and labor-intensive.

### 2.6 Non-traditional but well-establised methods

Manual X-ray evaluation, although a more advanced non-destructive technique, still depends heavily on human interpretation of radiographs to detect internal defects such as emptiness, cracks, or malformed embryos, which introduces subjectivity and limits throughput. MRI and X-ray are well-established but not traditional methods; they represent early generations of non-destructive imaging technologies.

As genebanks and breeding programs increasingly require scalable, high-resolution phenotyping, manual dimension measurements are being complemented or replaced by automated imaging systems. These systems extract size, shape, and morphological features rapidly and non-destructively across thousands of seeds, offering far richer datasets. Collectively, traditional approaches remain valuable reference methods in genebanks and seed testing laboratories, but their destructive nature, operator dependency, and low scalability highlight the need for automated, high-throughput phenotyping technologies that can deliver consistent and objective assessments across large seed collections.

## 3 Using the electromagnetic spectrum for seed phenotyping

**Figure 1:**
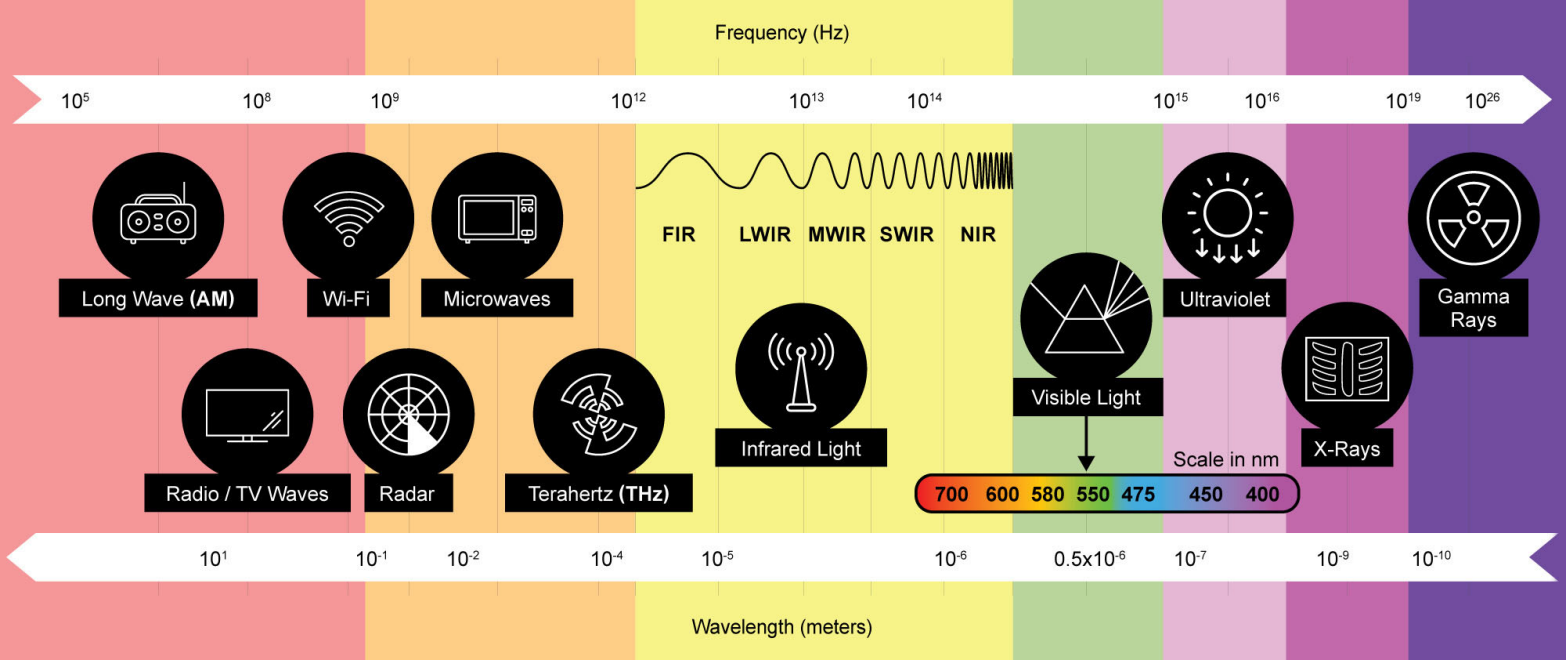
The electromagnetic spectrum.

The electromagnetic spectrum encompasses a range of electromagnetic waves, classified by their wavelengths/frequencies, from gamma rays to radio waves (**Figure 1**). Each range of the spectrum interacts uniquely with matter, making it a versatile tool for analyzing seed and plant traits in genebanks. By leveraging various regions of the spectrum, phenotyping technologies can non-invasively assess physical, physiological, and biochemical traits, enhancing the efficiency and precision of seed characterization (**Table 1**).

**Table 1:** Electromagnetic range and their use in seed phenotyping.

Electromagnetic tool	Wavelengths (m)	Current or potential use
Gamma rays	< 10^-10^	Create mutations in seeds
X-rays	2 × 10^-10^ – 2 × 10^-9^	See-through Seed embryo and structure
Ultraviolet	8 × 10^-9^ – 1.1 × 10^-7^	Activation of seed chemicals Seed sterilisation
Visible light	4 × 10^-7^ – 7 × 10^-7^	Seed shape, size, color Seed surface and visual traits
Near Infrared (NIR)	7 × 10^-7^ – 10^-6^	Seed water status
Shortwave infrared (SWIR)	10^-6^ – 2.7 × 10^-6^	Seed biochemical makeup Seed Nutrient content
Mediumwave infrared (MWIR)	3 × 10^-6^ – 8 × 10^-6^	Specific molecular vibrations Seed germination/vigor
Longwave infrared (LWIR)	8 × 10^-6^ – 1.4 × 10^-5^	Seed Thermal conductance Seed germination/vigor
Far infrared (FIR)	1.4 × 10^-5^ – 2.7 × 10^-5^	Seed and environmental heat dissipation Seed energy balance
Terahertz (THz)	3 × 10^-5^ – 1.2 × 10^-3^	Seed water distribution Seed stress responses
Shortwave microwaves	1.2 × 10^-3^ – 1.2 × 10^-2^	Seed moisture Seed structure
Radar (longwave microwave)	1.2 × 10^-2^ – 1.2 × 10^-1^	Seed structure
Wi-Fi	1.2 × 10^-1^ – 1.5 × 10^-1^	See through Seed structure
Radio waves	5 × 10^-1^ – 10	Seed viability & germination Seed moisture

Shorter wavelengths, such as gamma rays and X-rays, penetrate deep into seeds and are particularly useful for internal structural analysis. Gamma rays have historically been employed to induce mutations for breeding programs (Beyaz and Yildiz, 2017), while X-rays provide high-resolution imaging to evaluate embryo integrity and detect cracks or voids within seeds (Indore et al., 2022; Hamdy et al., 2024). Ultraviolet (UV) light, situated just beyond the visible spectrum, is utilized for sterilization purposes and chemical activation processes, offering applications in seed treatment and pathogen control (Arcos-Limiñana et al., 2025) (**Figure 1**) as well as general assessment of seed integrity.

In the visible light range, RGB imaging is widely used to capture basic physical traits like color, size, and shape. When the light used is a laser, reflectance imaging can also provide information on the viability of the seeds with a precision of the order of magnitude of the wavelength of the laser used (Zdunek et al., 2014). Expanding into the near-infrared (NIR) and shortwave infrared (SWIR) regions, hyperspectral and multispectral imaging detect subtle variations in biochemical composition, such as moisture, protein, and carbohydrate content (ElMasry et al., 2019), as well as signs of disease or stress in seeds (Vrešak et al., 2016) and structural information. Moving further along the spectrum, thermal infrared imaging detects heat signatures associated with metabolic activity, making it invaluable for assessing seed vigor and germination potential (Kranner et al., 2010) (**Figure 1**).

Longer wavelengths, including terahertz (THz) (Ghamkhar, 2023; Wang et al., 2024) and radio waves (Zhong et al., 2021), provide emerging possibilities for seed phenotyping at genebanks. Terahertz imaging offers non-invasive analysis of internal seed structures (Hui et al., 2018), moisture distribution (Fu et al., 2024) and early stress detection (Gua et al., 2013; GE et al., 2014; Ghamkhar, 2023) while radio waves, particularly through Wi-Fi imaging, present potential for real-time monitoring of seed health during storage and transport. These technologies are particularly appealing for their ability to penetrate layers and provide data without damaging seeds.

By utilizing the electromagnetic spectrum, genebanks can capture a comprehensive range of seed traits, from physical integrity to genetic and biochemical properties. The diverse applications of these technologies ensure that genebanks can maintain high-quality germplasm collections, enabling efficient conservation and utilization for breeding and agricultural innovation.

## 4 Sensor technologies for non-destructive seed phenotyping

Phenomics has emerged as a transformative approach for enriching genebank data, enabling precise, high-throughput assessment of seed and plant traits. Traditional genebank evaluation methods often relied on labor-intensive, time-consuming processes that limited the scope of characterization. By integrating advanced imaging and sensor technologies, phenomics provides scalable and automated solutions that generate comprehensive datasets, essential for breeding (Dwivedi et al., 2020; Ghamkhar, 2023), conservation (Lürig et al., 2021; Fernando et al., 2023), and research (Fernando et al., 2023). These advancements allow genebanks to assess large collections efficiently while offering deeper insights into the traits that underpin agricultural productivity and resilience.

### 4.1 Optical and imaging sensors

One of the most significant contributions of phenomics is the adoption of sensor technologies across the electromagnetic spectrum. Visible light (RGB) imaging is the standard digital imaging in the visible spectrum. It can be used to analyze seed morphology, color, and texture as well as for seed purity monitoring and detection of seed contamination. RGB imaging is Inexpensive and easy to implement and can be integrated into automated systems for high-throughput analysis. The downside of RGB imaging is its limitation to physical traits only. RGB imaging captures high-resolution images of seeds to assess seed physical traits (Lurstwut and Pornpanomchai, 2011; Mahajan et al., 2015; Zhang et al., 2018). By employing advanced algorithms, RGB imaging systems can automate the measurement of individual seed dimensions and classify them based on uniformity and appearance (Punthumast et al., 2012; Morales et al., 2024).

In addition to RGB imaging, multispectral and hyperspectral imaging provide enhanced capabilities for analyzing physical seed traits (ElMasry et al., 2019; Mortensen et al., 2021). These tools capture data across multiple wavelengths, allowing for the detection of subtle variations in surface texture, color intensity, and potential physical damage (Feng et al., 2019; Gaikwad and Tidke, 2022). Multispectral imaging is widely used for assessing physical seed traits such as size, shape, and color, which are critical for determining uniformity and purity (ElMasry et al., 2019, ElMasry et al., 2020; Li et al., 2021; Tu et al., 2021). Hyperspectral imaging, which captures data across a broad range of wavelengths, is particularly valuable for detecting biochemical markers linked to seed health, genetic purity, and quality traits such as protein content (Dumont et al., 2015; Ambrose et al., 2016a) or disease presence (Dumont et al., 2015; Lee et al., 2017). These tools provide unparalleled accuracy and resolution, enhancing the reliability of seed characterization (see section…. below).

### 4.2 3D and structural imaging technologies

Tools and approaches such as 3D imaging technologies are used to capture seed structural traits (Jahnke et al., 2016), while CT scanning allows for detailed internal seed analysis (Gargiulo et al., 2020; Kunishima et al., 2020). By combining these technologies, phenomics not only enriches genebank datasets but also facilitates the prioritization of accessions with traits like nutritional quality, ensuring that genebanks remain vital resources in addressing global agricultural challenges.

The 3D imaging technique uses structured light or laser scanning to create 3D models of seeds (Huang et al., 2022; Qin et al., 2022). Genebanks can use this method to measure seed size, shape, and volume accurately. It is also a good tool to assess seed uniformity and detect physical defects. 3D images are suitable for morphological characterization and trait standardization and enable digital archiving of seed phenotypes. If genebanks choose 3D imaging for seed screening, they will require significant data storage and processing capabilities.

### 4.3 X-ray and wi-fi imaging tools for embryological and structural analysis

X-ray imaging has become an indispensable tool for non-invasive structural analysis of seeds, offering insights into embryological health and potential physical damage (Arkhipov et al., 2019). X-ray imaging complements RGB and spectral imaging methods by enabling non-invasive internal structural analysis, such as identifying cracks, voids, or malformed embryos within seeds (Xia et al., 2019; França-Silva et al., 2023). X-ray imaging further complements spectral imaging techniques by assessing structural integrity, which correlates with physiological performance (Arkhipov et al., 2019; Medeiros et al., 2020). This technology allows genebanks to assess seed viability by visualizing internal structures, such as the embryo and endosperm, without the need for destructive sampling. X-rays are particularly effective in identifying cracks, voids, or malformations within the seed, ensuring the preservation of valuable germplasm. The ability to analyze seed batches rapidly and accurately makes X-ray imaging a vital component of quality control in genebanks, enabling researchers to maintain seed integrity and optimize storage strategies.

X-ray creates detailed images of seed internal structures, helping identify physical damage or abnormalities caused by microbial infestations (Arkhipov et al., 2019; Nadimi et al., 2022). ​It helps the user to evaluate internal seed morphology (e.g., embryo and endosperm development) (Silva et al., 2013; Ahmed et al., 2018). Detecting physical damage, voids, or abnormalities as well as assessment of seed viability and vigor indirectly through structural health are other enablers of genebanks (Whitehouse et al., 2020). Additionally, X-ray imaging is useful for assessment of the embryological status, allowing the identification of seeds with optimal endosperm and embryo development. This non-invasive, reliable and easy to automate technique is very suitable for high-throughput analysis of genebank data. Phase-contrast X-ray provides contrast not only on dry sample but also on wet samples (Rousseau et al., 2015). On the downside, safety protocols are needed for handling X-ray equipment.

Wi-Fi imaging, a more recent development, represents a novel approach to structural and biochemical seed analysis (Roitsch et al., 2019). By utilizing microwave frequencies, Wi-Fi imaging has the potential to penetrate seed layers to detect internal anomalies and moisture distribution. This technology is less resource-intensive than methods like MRI, making it a cost-effective alternative for genebanks with limited budgets. Wi-Fi imaging also holds promise for real-time monitoring of seed health during storage and transport, offering dynamic insights into changes in seed composition over time. While X-ray imaging is a much more mature and ready-to-use technology, together, X-ray and Wi-Fi imaging push the boundaries of what is possible in embryological and structural analysis, providing genebanks with cutting-edge tools to ensure the longevity and quality of their collections.

### 4.4 Spectroscopy-based sensors

Phenomics tools play a crucial role in ensuring seed genetic purity by providing high-throughput, non-destructive methods for evaluating seed traits. These tools utilize advanced technologies like near-infrared (NIR) spectroscopy, and machine vision to detect subtle phenotypic variations that indicate genetic differences. This spectroscopy tool has been in play for decades now and uses the near-infrared region of the spectrum to assess chemical properties of seeds (Fassio and Cozzolino, 2004; Font et al., 2006). Genebanks can use this method to detect seed quality traits such as protein, oil and starch content as well as seed moisture and contaminants (Agelet and Hurburgh, 2014; Reddy et al., 2022). This rapid and non-destructive technique is suitable for high-throughput seed screening and analysis although it requires calibration models specific to seed types (Baye et al., 2006; Font et al., 2006).

Fourier-transform infrared (FTIR) spectroscopy and Raman spectroscopy are powerful vibrational spectroscopic techniques that enable rapid, non-destructive characterization of water content and chemical composition in seeds (Ambrose et al., 2016b). FTIR measures the absorption of infrared light associated with molecular vibrations, particularly those involving O–H, C–H, C=O, and N–H bonds (Berthomieu and Hienerwadel, 2009). Because water molecules exhibit strong and distinct IR absorption peaks, FTIR provides a sensitive means of quantifying seed moisture and monitoring hydration dynamics during imbibition or storage (Upretee et al., 2024). Additionally, FTIR can detect lipids, proteins, carbohydrates, and secondary metabolites through their characteristic spectral signatures (Hssaini et al., 2021). With appropriate chemometric modelling, FTIR has been used to estimate oil content, protein levels, seed ageing status, and other quality traits across a wide range of crop species (Rahman and Cho, 2016). Its high throughput, minimal sample preparation, and ability to analyze intact seeds make it highly suitable for genebank and breeding applications (Aftab et al., 2025).

Raman spectroscopy, in contrast, measures inelastic scattering of monochromatic light (usually from a laser) to provide a complementary molecular fingerprint that is less affected by water (Smith and Dent, 2019). This makes Raman particularly valuable for studying internal biochemical changes in seeds without interference from moisture, enabling reliable assessment of oil composition, carotenoids, phenolics, and other chemically informative compounds (Sanchez et al., 2019). Raman microscopy can also resolve spatially localized biochemical differences, such as the distribution of storage proteins or lipids within specific seed tissues, offering insights that bulk methods cannot provide (Saletnik et al., 2021). Recent advances in handheld Raman systems and surface-enhanced Raman spectroscopy (SERS) have further expanded its potential for rapid seed screening, including early detection of seed ageing, pathogen contamination, or varietal identity (Weng et al., 2021).

Together, NIR, FTIR and Raman spectroscopy represent versatile tools for modern seed phenotyping by providing rapid, high-resolution chemical profiling without compromising seed viability. These techniques allow researchers and genebanks to move beyond traditional destructive assays toward scalable, predictive, and data-rich approaches to seed quality assessment (Xia et al., 2019). Their integration with machine learning enables automated classification of viability, moisture status, or compositional traits, positioning all three technologies as cornerstones for future high-throughput phenotyping platforms (Kolašinac et al., 2025).

### 4.5 Hyperspectral imaging

multispectral and hyperspectral imaging provide valuable insights into seed vigor and stress tolerance by detecting biochemical markers such as chlorophyll, carotenoids, and proteins (Wakholi et al., 2018; ElMasry et al., 2019). These markers are indicative of a seed's physiological state and its capacity to withstand adverse environmental conditions (Bewley and Black, 2012; Corbineau, 2012). For example, hyperspectral imaging can detect early signs of stress by identifying spectral changes associated with oxidative damage or nutrient deficiencies. HSI is also used to assess seed composition (e.g., protein, starch, oil, or moisture content) (Dumont et al., 2015; Ambrose et al., 2016a) and potentially help in understanding structure of seeds. It can also be used to detect seed viability, health, and damage (e.g., fungal infections, mechanical damage) (Feng et al., 2019; Ferreira et al., 2024). It can differentiate between seed types and varieties, too (Tan et al., 2019; Zhu et al., 2019). The non-destructive and highly precise nature of this technology along with providing both physical and chemical data in a single scan are the advantages of HSI. The technology requires expertise in data analysis and interpretation and high data storage capacity.

Hyperspectral and multispectral imaging are among the most effective tools for analyzing seed content (Su and Sun, 2018), as they capture spectral data across a wide range of wavelengths, enabling the detection of biochemical markers like proteins, starch, lipids, and antioxidants. For instance, hyperspectral imaging can non-invasively predict amylose content in grains or identify seeds with high oil content (Mahajan et al., 2015; Akewar and Chandak, 2023), providing valuable insights for both breeding and quality control. Similarly, these tools can detect moisture content variations, which are critical for determining seed storability and germination potential (Mahajan et al., 2015; Wang et al., 2021). Multispectral and hyperspectral imaging are widely employed to detect subtle spectral differences associated with seed infections or contamination (Jaillais et al., 2015; Feng et al., 2019). These tools can identify early signs of fungal or bacterial pathogens by analyzing specific wavelengths that correspond to biochemical changes caused by disease (Olesen et al., 2011; da Silva et al., 2021; Singh et al., 2021). HSI also shows biochemical and morphological differences between genetically pure seeds and contaminants (ElMasry et al., 2019; Dhanya et al., 2024).

### 4.6 Magnetic resonance imaging

This technology uses magnetic fields and radio waves to visualize internal seed structures (Foucat et al., 1993; Tuomainen et al., 2022). MRI would be the perfect tool to study water content and movement in seeds during imbibition and germination (Terskikh et al., 2005; Garnczarska et al., 2007). It can also be used to assess seed dormancy and viability (Gruwel et al., 2002). The advantage of using MRI in genebanks is its high resolution, non-invasive internal imaging capacity but its high cost and complexity make its adoption difficult for genebanks.

### 4.7 Thermal, fluorescence, and novel sensors

Thermal (Kim et al., 2013; Men et al., 2017) and terahertz imaging (GE et al., 2014; Hu et al., 2024a) extend these capabilities by evaluating physiological qualities like germination capacity, vigor, and moisture content. Technologies like transcriptome analysis and epigenomics complement phenotyping by identifying genetic and functional variations associated with traits such as seed longevity, stress tolerance, and germination. For example, studies on DNA methylation have revealed its role in germination and viability (Mira et al., 2020), particularly in recalcitrant seeds (Ge et al., 2025), while metabolomics has been instrumental in predicting seed quality traits like amylose content and biochemical composition (Wang et al., 2022; Hu et al., 2024b). These advancements bridge the gap between phenotypic observations and underlying genetic mechanisms, providing actionable insights for crop improvement.

Thermal imaging is a key tool for measuring metabolic activity during seed germination, as it detects heat signatures associated with water uptake and respiration processes (Kranner et al., 2010; ElMasry et al., 2020). By analyzing temperature variations, researchers can quickly identify viable seeds and predict germination rates (Men et al., 2017). Infrared imaging (IRI) measures the heat emitted by seeds (Kranner et al., 2010; ElMasry et al., 2020). Using this system enables the genebanks to monitor seed water content and its drying process while also detecting metabolic activity in seeds during germination tests (Xia et al., 2019). The IR cameras also help in assessment of seed viability and stress responses. The non-destructive and sensitive IR helps detect metabolic changes early and at seed stage. It should be considered that the use of IR is only limited to germination and drying studies though.

Thermal imaging also detects temperature variations caused by metabolic activity, which can indicate the presence of pathogens or pests within seeds (Liu et al., 2020; Wen et al., 2023).

Fluorescence imaging technology detects fluorescence emitted by seeds after exposure to specific light wavelengths (Barboza da Silva et al., 2021; Bartolić et al., 2022). It can be used to identify seed viability and vigour by detecting metabolic compounds (e.g., chlorophyll or secondary metabolites) (Galletti et al., 2020; Batista et al., 2022). This imaging technique also enables the genebanks to assess seed health, including fungal or pest contamination (Pasikatan and Dowell, 2001; Bartolić et al., 2022). Fluorescence imaging is highly sensitive to physiological and biochemical changes. This technique may require specialized equipment and expertise. Chlorophyll fluorescence can also reveal metabolic differences linked to genetic variation helping seed genetic purity detection.

Ultrasonic sensors use sound waves and can be used to probe the physical properties of seeds (Sattar and Laila, 2025). These sensors can detect internal voids or fractures in seeds and help genebanks assess seed hardness and physical integrity in a rapid and non-destructive manner. However, this technique is less common and may require development for specific applications.

Terahertz time-domain spectroscopy (THz-TDS) is emerging as a powerful non-destructive tool for probing the internal physical and chemical properties of seeds, offering unique capabilities that complement optical and infrared methods. Terahertz imaging enables the assessment of internal moisture distribution, a critical factor for both germination and longevity (Lei et al., 2022; Hu et al., 2024a). Operating in the 0.1–10 THz frequency range, THz-TDS measures the time-resolved transmission or reflection of ultrafast terahertz pulses as they interact with seed tissues (Rawson and Sunil, 2022). Because terahertz waves are highly sensitive to polar molecules—particularly water—as well as structural discontinuities such as voids, cracks, and embryonic defects, THz-TDS can reveal information related to seed moisture content (Ge et al., 2023), density, and internal integrity (Afsah-Hejri et al., 2019). Its strong response to bound and free water makes it especially suited for characterising hydration dynamics, monitoring desiccation processes (Pal and Chattopadhyay, 2021), and assessing early viability loss associated with membrane deterioration or changes in internal microstructure.

Although still developing as an applied technology in seed science (Jiang et al., 2024), advances in compact terahertz sources, improved signal acquisition, and machine-learning-based spectral interpretation are rapidly increasing its practicality. As a result, THz-TDS is poised to become an important addition to the non-invasive phenotyping toolbox for genebanks and seed researchers aiming to evaluate seed quality at scale.

Advanced imaging technologies, such as THz imaging and thermal imaging, further enhance seed content quality analysis (ElMasry et al., 2020; Ge et al., 2021). Terahertz imaging enables non-invasive assessment of storage-related degradation (Han et al., 2024; Li et al., 2025). Further, THz scans detecting anomalies may signify genetic impurities in seeds.

## 5 Seed quality traits measurable through sensor technologies

Seed quality traits are critical indicators of the viability, vigor, and utility of seeds for agricultural and conservation purposes (De Vitis et al., 2020; Gough, 2020). These traits can be broadly categorized into physical, physiological, health, and content traits, each playing a pivotal role in determining the overall performance of seeds. Physical traits, such as size, shape, color, and uniformity, are essential for ensuring mechanical handling efficiency and market acceptability (McDonald and Panozzo, 2023). For instance, uniformity in seed size facilitates precision sowing and promotes even germination (Finch-Savage and Bassel, 2016). No damage to the external parts of seeds is a crucial physical quality trait (Lemes and Catão, 2024). Analytical purity, or the absence of contamination by weed seeds, other crops, or inert matter, is another vital physical trait, ensuring the integrity of seed lots for planting and trade.

Physiological traits focus on the regenerative capacity of seeds, encompassing germination rate, vigor, and longevity. Germination rate measures the ability of seeds to sprout under optimal conditions, while vigor assesses their potential to thrive under suboptimal or stressful environments (Finch-Savage and Bassel, 2016; Reed et al., 2022). Longevity, or the capacity of seeds to retain viability over extended storage periods, is especially critical for genebanks and seed conservation programs (Walters et al., 2005; Kameswara Rao et al., 2017). Together, these traits ensure seeds not only germinate but also establish healthy plants that can withstand environmental stresses.

In addition, health and content quality traits are paramount for maintaining the productivity and reliability of seed stocks (Kameswara Rao et al., 2017; Sundareswaran et al., 2023). Health traits, such as being disease-free and having optimal moisture content, directly affect storage stability and field performance (Kameswara Rao et al., 2017; Kumar et al., 2023). Content traits, including biochemical and embryological as well as microbiome composition may help the planted seeds produce crops with predictable and desirable characteristics.

Further, genetic traits such as purity and the absence of undesirable mutations are important for resistance to pests, environmental stressors, and enhanced nutritional profiles (Singh et al., 2015) but these quality traits can be screened using genomics tools. The holistic evaluation of these seed quality traits allows genebanks, breeders, and farmers to optimize seed utilization, supporting food security and sustainable agriculture.

### 5.1 Seed physical quality traits

Phenomics tools have revolutionized the measurement of seed physical quality traits by enabling rapid, precise, and non-destructive assessments (Ghamkhar, 2023; Hacisalihoglu and Armstrong, 2023). Physical traits such as size, shape, density, color, and uniformity are fundamental to seed quality, influencing mechanical handling, market value, and sowing efficiency Phenomics technologies ensure the consistent grading of seed lots, improving quality control processes in both genebanks and commercial seed production.

The integration of these phenomics tools not only enhances the efficiency of physical trait evaluation but also ensures the preservation of seed samples for conservation and research purposes. By combining accuracy, speed, and non-destructive methodologies, phenomics technologies play a critical role in maintaining and enhancing seed physical quality standards (Dell'Aquila, 2007; Mahajan et al., 2015).

Another important physical quality trait is seed analytical purity, i.e. no weeds or other crop seeds in the lot (Powell, 2009; Prasad, 2023). RGB imaging (using different filters) (Granitto et al., 2005), multi- and hyper-spectral imaging and thermal imaging can all help in high throughput phenotyping for this important factor/trait at both genebanks and commercial seed exchange.

### 5.2 Seed physiological quality traits

Phenomics tools have greatly advanced the assessment of seed physiological quality traits, providing non-invasive and precise methodologies to evaluate attributes like germination potential, moisture content, vigor, and viability (Al-Amery et al., 2018; Feng et al., 2019). These traits are essential for understanding a seed’s regenerative capacity and its ability to establish healthy plants under both optimal and stressful conditions (Finch-Savage and Bassel, 2016; Reed et al., 2022).

By integrating phenomics tools, researchers and genebanks can effectively evaluate the physiological quality of seeds, ensuring the preservation of high-performing germplasm.

### 5.3 Seed pathology quality traits

Seed pathology quality (infected vs. healthy seeds) is a critical parameter for ensuring the longevity of germplasm, as well as the success of agricultural production (Gebeyaw, 2020). Phenomics tools provide advanced, non-invasive methods to detect and evaluate health-related traits such as the presence of pathogens, disease resistance, and durability during storage (Walcott et al., 1998; Guelpa et al., 2015)– including hard seededness in legumes, which extends seed dormancy. This capability allows for rapid and precise disease detection, reducing the reliance on traditional, labor-intensive diagnostic methods.

Emerging technologies, such as cold plasma treatment, are being coupled with imaging systems to both detect and mitigate seed-borne pathogens (Mravlje et al., 2021; Moumni et al., 2023). These phenomics tools not only enhance the efficiency and accuracy of seed health evaluations but also support better decision-making for storage and planting, ensuring that only healthy, high-quality seeds are used and preserved (Yang et al., 2020; Dhanya et al., 2024).

Magnetic Resonance Imaging (MRI) is an expensive and slow tool and despite providing valuable information, it is not suggested as a phenomics tool for genebanks (Ghamkhar et al., 2024). RGB imaging alone is also not informative enough for seed health assessment as it will only provide information when it is already too late to separate healthy seeds from diseased ones (Vrešak et al., 2016). It also misses the intermediate stages between healthy and diseased seeds.

### 5.4. Seed biochemical quality traits

Phenomics tools have significantly advanced the evaluation of seed content traits, such as biochemical composition, nutrient profiles, embryological status, anti-nutritional factors and microbiome composition (Singh et al., 2020; Hacisalihoglu and Armstrong, 2023). These traits are critical for assessing seed viability, nutritional value, and suitability for specific agricultural or industrial applications.

Wi-Fi holography technology (Roitsch et al., 2019) and MRI (Plutenko et al., 2025) (more informative but expensive) are also new tools that enable embryo integrity screening while the seed microbiome can be evaluated using muti- hyper-spectral imaging (Reddy et al., 2023) (less informative but more affordable) systems.

By integrating these phenomics tools, genebanks and researchers can perform rapid, precise, and non-destructive evaluations of seed content traits, ensuring the preservation of high-value germplasm and the optimization for specific traits of interest in the seed collection.

### 5.5 Seed genetic purity traits

For example, hyperspectral imaging captures spectral signatures across various wavelengths, revealing These spectral patterns, combined with machine learning algorithms, allow for accurate classification and sorting of seeds based on purity traits, thereby enhancing quality assurance in breeding programs and seed production.

Meanwhile, Automated phenotyping platforms, integrated with AI and deep learning, can process large datasets to identify and predict genetic purity traits with high accuracy (Tu et al., 2021; Mukasa et al., 2022).

## 6 Key applications of phenomics in genebanks and seed research

**Figure 2:**
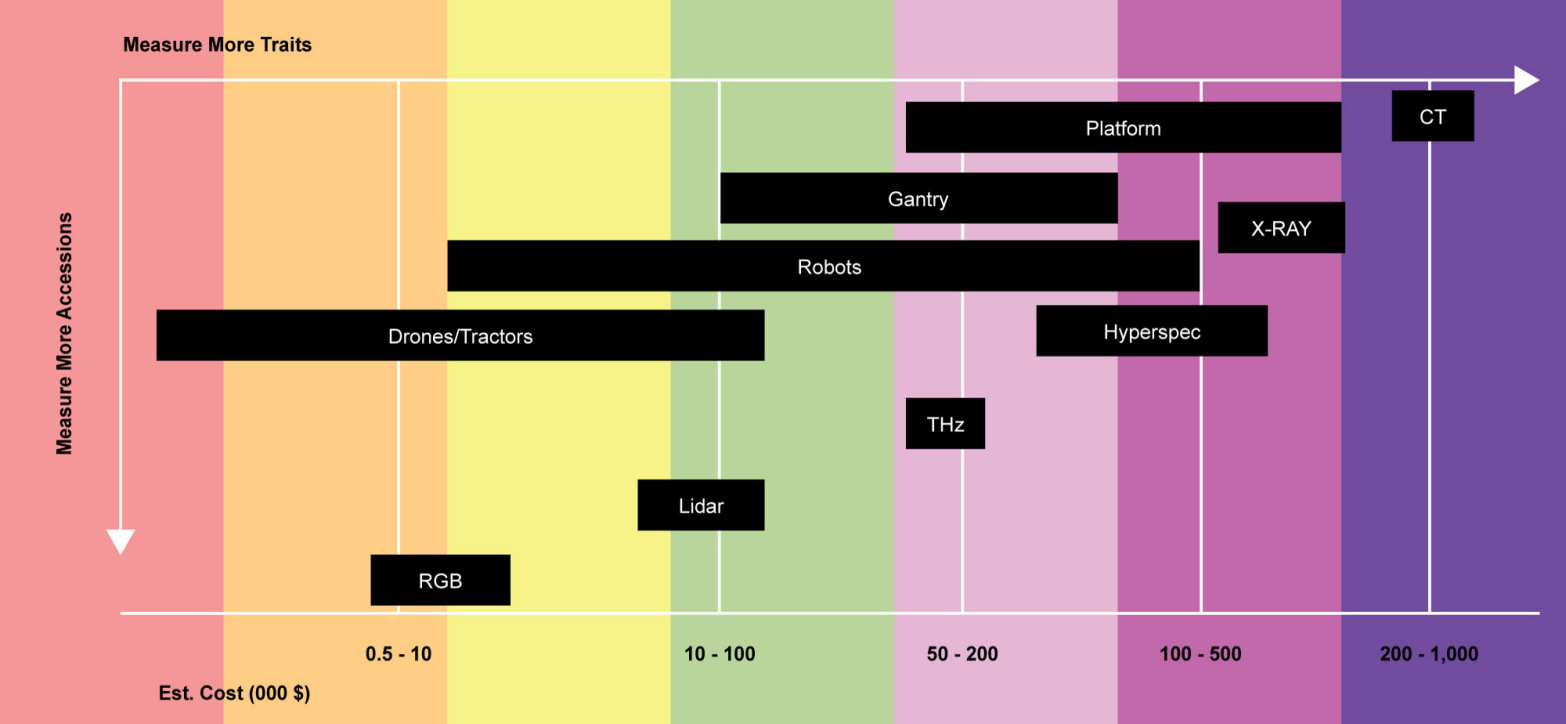
Estimated cost of a few sensor technologies in genebanks (includes the costs of data preparation but not data processing).

Genebanks, as custodians of plant genetic resources, can significantly enhance their seed phenotyping capabilities by adopting modern phenomics technologies. These technologies allow for non-destructive, high-throughput, and precise assessment of seed traits, ensuring better management, characterization, and utilization of genetic resources. Key sensor and imaging technologies that genebanks should adopt for seed phenotyping will largely depend on a cost/value assessment and whether a quick gains approach best suits genebanks. In general, there are some approaches currently available to genebanks with a range of costs (**Figure 2**). In the meantime, the aim should be to make efforts for collaboration in order for the availability and use of new and quick gain technologies for genebanks. Below are key sensor and imaging technologies that genebanks could adopt for seed phenotyping:

### 6.1. Seed viability scanning at scale

One of the most critical applications of phenomics at genebanks is in seed quality assessment, where phenomics platforms are used to evaluate physical, physiological, health, and genetic attributes (Volk et al., 2021; Ghamkhar, 2023). Physiological traits such as germination capacity, vigor, and longevity are measured using advanced imaging methods (Hu et al., 2024a; Jin et al., 2025). These tools ensure that only high-quality seeds are preserved and distributed for breeding and research purposes.

### 6.2 Detecting dormancy, ageing and storability

Detecting seed dormancy and ageing is a critical component of seed phenotyping, as these physiological states directly influence germination performance, storage behavior, and long-term viability within genebanks (Whitehouse et al., 2020). Seed dormancy—whether physiological, morphological, or physical—represents a controlled inhibition of germination (Nonogaki, 2014), and phenotyping efforts increasingly focus on identifying biochemical and structural markers that distinguish dormant from non-dormant seeds before testing. Traditional dormancy detection relies on germination assays under varying temperature, light, or chemical treatments (e.g., gibberellic acid or nitrate), but advances in sensing technologies now allow the detection of traits associated with dormancy-breaking or dormancy-imposing mechanisms. These approaches enable earlier and more accurate identification of dormancy-related traits without the need for extended germination trials.

Seed ageing detection focuses on identifying early biochemical and structural deterioration that precedes visible loss of viability (Xing et al., 2025). During seed ageing—whether natural or accelerated—changes occur in membrane integrity, lipid peroxidation levels, antioxidant capacity, protein carbonylation, and mitochondrial activity (Adetunji et al., 2021). These processes alter the optical, thermal, and dielectric properties of seeds, making them detectable through non-destructive phenotyping technologies. The integration of these approaches allows genebanks and seed scientists to monitor ageing trajectories more effectively, supporting predictive models of seed longevity and improving prioritization for regeneration.

Phenomics enhances the evaluation of seed health and storage performance, critical aspects of genebank operations (Ghamkhar, 2023). This real-time monitoring capability allows genebanks to address potential issues before they compromise seed viability, ensuring long-term preservation (Bhattacharya and Mummenhoff, 2024). These applications make phenomics indispensable for maintaining the quality and longevity of germplasm collections (Nguyen and Norton, 2020; Ghamkhar et al., 2024).

### 6.3. Pre-breeding characterization

Phenomics tools also play a vital role in trait discovery and association mapping (Hatzig et al., 2015; Xiao et al., 2022). By linking seed phenotypic data with genomic information, researchers can identify markers associated with complex traits such as biochemical composition (Chaudhary et al., 2015; Dwivedi et al., 2021). This capability is particularly valuable for breeding programs targeting the development of high-yield or nutrient rich crops (Gaikwad et al., 2020; Dwivedi et al., 2021). Additionally, trait discovery enables genebanks to identify and preserve rare or underutilized accessions with unique seed characteristics, ensuring their availability for future use (Smale and Jamora, 2020).

### 6.4 Rapid seed purity testing

Rapid seed purity testing is an essential component of modern seed and genebank management, enabling the quick verification of genetic, physical, and varietal integrity in seed lots destined for breeding, research, or commercial production (Gough, 2020). Traditionally, purity testing relied on manual/visual inspection, examining seeds for off-types, contaminants, or foreign botanical materials. This approach is labor-intensive, subjective, and limited by human detection capabilities. Advances in imaging and machine-learning technologies have transformed purity assessment by enabling high-throughput, objective, and non-destructive analysis of large seed batches. These approaches significantly reduce testing time, increase reproducibility, and enable the detection of subtle phenotypic differences that may not be visible to the human eye.

Beyond visual and spectral methods, molecular approaches such as DNA barcoding, SNP profiling, and PCR-based assays contribute to genetic purity verification. This is specifically the case in hybrid or certified seed production systems where varietal identity is critical. Emerging phenomics technologies offer additional layers of discrimination by detecting biochemical signatures associated with specific genotypes or contaminants.

### 6.5 Automating regeneration decisions

Automating regeneration decisions is becoming a transformative application of seed phenotyping technologies, offering genebanks a data-driven way to prioritize which accessions require regeneration and when (Nguyen and Norton, 2020). Traditionally, regeneration choices have relied on simple viability thresholds obtained from periodic germination tests, often carried out on fixed schedules (e.g., every 5–10 years). If seed viability falls below 85% of the initial germination percentage in active collections regeneration is required (Dulloo et al., 2008). While effective, this approach can be inefficient as viability may remain high for decades in some species, while others deteriorate rapidly and risk genetic loss before the next scheduled test. Automated decision-support systems integrate phenotyping outputs such as germination behavior, ageing biomarkers, seed moisture profiles, and imaging-derived structural or biochemical indicators to predict viability decline more precisely (Angidi et al., 2025). By combining these measurements with models of seed longevity, storage environment data, and historical regeneration records, genebanks can generate real-time risk assessments and dynamic prioritization lists that minimize unnecessary regeneration while preventing loss of valuable genetic diversity.

Machine-learning algorithms and predictive analytics further strengthen this automation by identifying patterns that link phenotypic traits to longevity outcomes, enabling early detection of ageing, before viability visibly declines (Jin et al., 2025). High-throughput phenotyping tools can screen hundreds of accessions rapidly, feeding multi-dimensional data into longevity prediction models. These systems can alert managers when accessions are approaching critical thresholds or when specific seeds exhibit atypical ageing trajectories. Automating regeneration decision enhances operational efficiency, reduces costs, and ensures the timely regeneration of high-value or vulnerable germplasm. Ultimately, this approach enables genebanks to maintain collections more strategically, preserving genetic integrity while optimizing resource use across large and diverse seed repositories.

### 6.6 Linking phenotype–genotype in digitized gene bank workflows

Historically, genebanks have maintained extensive passport and storage information but comparatively sparse phenotypic and genomic datasets (Anglin et al., 2018). Modern digitization efforts—coupled with advances in high-throughput phenotyping and affordable sequencing—now make it possible to systematically connect seed traits with underlying genetic variation across thousands of accessions. Integrating genomics with phenomics has significantly enhanced data enrichment in genebanks, providing a deeper understanding of seed and plant traits at both the molecular and phenotypic levels (Schmutzer et al., 2017; Ghamkhar et al., 2024). This integration not only improves the characterization of genetic resources but also facilitates the identification of genes associated with specific adaptive or agronomic traits (Volk et al., 2021; Ghamkhar et al., 2024).

The fusion of genomics and phenomics also supports predictive modeling and trait discovery (Togninalli et al., 2023). By correlating genetic data with high-throughput phenotypic measurements, genebanks can generate multidimensional datasets that reveal complex trait networks, such as those related to biochemical composition (Volk et al., 2021; Xiao et al., 2022). These enriched datasets are critical for tackling global agricultural challenges, such as food security. The seamless integration of genomics with phenomics in genebanks not only enhances the depth and reliability of germplasm evaluations but also accelerates the development of crop varieties tailored to future environmental and market demands (Egan and Stiller, 2022; Crossa et al., 2024).

Imaging-derived phenotypes (e.g., seed size, color, density, biochemical signatures), germination and ageing profiles, and environmental metadata can be integrated with genomic information such as SNP markers, whole-genome sequences, or genotyping-by-sequencing outputs. This creates a unified digital framework where each seed lot is represented not only by its identity and origin, but also by quantifiable traits and genetic markers that explain adaptive patterns or functional differences.

Embedding these phenotype–genotype linkages directly into genebank information systems enables powerful new workflows (Arend et al., 2022). Phenotypic screening data from seed imaging or physiological tests can help target genomic analyses toward accessions displaying unique or extreme characteristics. These integrated datasets also support the development of core collections and pre-breeding pipelines, thereby increasing the value and utilization of conserved germplasm.

Ultimately, linking phenotype and genotype in digitized genebank workflows enhances conservation decisions, improves the efficiency of regeneration and characterization strategies, and strengthens the role of genebanks as central hubs for innovation in seed science and plant improvement.

## 7 Challenges and opportunities in using seed phenomics at genebanks

Genebanks are and will be facing many challenges (and opportunities) if they proceed to integrating seed phenomics to their management system. A list of these challenges and opportunities is itemised below although it is by no means comprehensive.

### 7.1. Large imaging datasets

Large imaging datasets present both a significant challenge and a transformative opportunity for genebanks integrating seed phenotyping technologies. The vast amount of data generated by high-throughput phenotyping systems poses significant challenges for data storage, processing, and analysis (Shakoor et al., 2017; Yang et al., 2020). Effective data management frameworks and machine learning algorithms are required to ensure that the data generated is actionable and supports decision-making in conservation programs (Yu and Chung, 2024). There is a lot that can be learnt from the biomedical sciences in this space (Alkhatib and Gaede, 2024). Managing these datasets requires robust data infrastructure, including scalable storage solutions, standardised metadata formats, and efficient pipelines for image processing and annotation. Without these systems in place, genebanks risk accumulating unusable data backlogs, where images exist but cannot be analysed, interpreted, or linked to accession records.

At the same time, these large imaging datasets open unprecedented opportunities for enhancing genetic resource conservation and utilisation. These quantified imaging datasets enable machine-learning models to predict traits such as dormancy status, ageing progression, or embryo integrity at scale, dramatically increasing the throughput and precision of seed evaluations. Moreover, image archives can be re-analysed as new algorithms and computational tools emerge, allowing genebanks to extract additional value from historical phenotyping efforts.

### 7.2 Lack of shared standards

Another challenge lies in the standardisation of phenomics protocols and data management (Houle et al., 2010; Krajewski et al., 2015). Currently, different genebanks use diverse imaging platforms, resolutions, lighting conditions, trait definitions, and analytical pipelines. With a wide array of phenotyping tools and platforms available, there is often inconsistency in how traits are measured and reported across genebanks (Anglin et al., 2018; Nguyen and Norton, 2020). This lack of standardisation can hinder the comparability and integration of data, limiting the utility of phenomics in global collaboration efforts (Großkinsky et al., 2015; Awada et al., 2024). Without standardised trait ontologies, calibration procedures, and reporting guidelines, phenotypic data cannot be reliably integrated into global information systems or used to support large-scale meta-analyses. The absence of common data formats and quality thresholds also complicates long-term data curation and interoperability with existing genebank databases such as GRIN, Genesys, or national PGR information systems. As a result, valuable phenotyping outputs risk becoming siloed, underutilised, or incompatible with the broader plant genetic resources community.

Despite these challenges, the current lack of shared standards also presents a unique opportunity for the genebanking community to take a leadership role in establishing coordinated, future-proof frameworks for digital phenotyping. By collaborating through international networks, such as the Global Crop Diversity Trust, EURISCO, CGIAR genebanks, International Seed Testing Association (ISTA), and the International Plant Phenotyping Network, institutions can co-develop standard operating procedures (SOPs), trait ontologies, metadata requirements, and reference materials tailored specifically to seed phenotyping. Establishing shared benchmarks for image quality, sensor calibration, and data annotation will enable cross-genebank comparability and facilitate the exchange of validated protocols. Over time, this harmonisation will reduce duplication of effort, improve data reusability, and enable federated machine-learning models trained on multi-genebank datasets. Ultimately, addressing the lack of shared standards is not just a technical requirement but a strategic opportunity to build a globally cohesive, interoperable, and scientifically rigorous foundation for next-generation seed phenotyping.

### 7.3 Cost and instrument complexity

The adoption of phenomics tools in genebanks presents a range of challenges, particularly related to the cost and scalability of these technologies (Nguyen and Norton, 2020; Ghamkhar et al., 2024). Advanced platforms such as hyperspectral scanners, micro-CT systems, terahertz spectroscopy units, and automated imaging chambers require substantial capital investment, ongoing maintenance, and specialised technical expertise. For many genebanks, especially smaller national collections or those in resource-limited regions, these costs are prohibitive, limiting their ability to modernise evaluation workflows or transition from manual to digital phenotyping (Ghamkhar, 2023; Ghamkhar et al., 2024). Additionally, complex instruments often require trained operators, dedicated analysis software, and regular calibration, all of which introduce operational bottlenecks (Nguyen and Norton, 2020; Ghamkhar et al., 2024). This can lead to uneven technological capacity across genebanks, with only a few well-funded centers able to conduct high-throughput or high-resolution phenotyping, ultimately constraining global data integration and interoperability. This highlights the need for affordable and user-friendly solutions that can be deployed across diverse operational contexts.

However, these challenges also create opportunities for innovation, collaboration, and strategic investment. As sensor technologies mature, costs are gradually decreasing, and simpler, more compact instruments, such as portable NIR devices, low-cost multispectral cameras, or benchtop X-ray units, are becoming increasingly accessible (Argirusis et al., 2025). These emerging tools can serve as stepping stones for genebanks to develop digital phenotyping capabilities without immediately committing to high-end systems. Furthermore, shared infrastructure models, such as regional phenotyping hubs, cross-institutional equipment pools, cloud-based analysis environments, and collaborative service agreements, allow multiple genebanks to access advanced technologies without bearing the full financial burden individually. Over time, strategic investment and coordinated capacity building can turn cost and complexity from limiting factors into catalysts for modernisation, equity, and innovation in global seed conservation.

### 7.4 Calibration drift and transferability

Instruments such as multispectral and hyperspectral imagers, X-ray systems, and terahertz platforms rely heavily on stable calibration to ensure consistent and reproducible measurements. Over time, however, sensor ageing, environmental fluctuations, lamp degradation, and hardware servicing can introduce calibration drift, leading to subtle but significant measurement inconsistencies. For genebanks, where phenotyping may occur over years or decades and where data must remain comparable across regeneration cycles such drift can compromise longitudinal analyses, trait prediction models, and digital records associated with accessions. Similarly, the transferability of calibration models between instruments, laboratories, or organisations is often limited: a predictive model built on one device or platform may fail or perform poorly when applied to another due to differences in optics, illumination, detector sensitivity, or spectral resolution. This limits the scalability of phenotyping workflows and poses a barrier to harmonised global datasets.

Calibration drift and transferability issues also offer opportunities to develop more robust and collaborative phenotyping ecosystems. Regular calibration schedules and the implementation of automated self-calibration routines can improve long-term instrument stability in genebanks. Emerging machine learning approaches, such as domain adaptation, instrument standardisation algorithms, and transfer learning (Chato and Regentova, 2023), can significantly enhance model portability across devices and institutions. Collaborative calibration frameworks, where multiple genebanks collect reference datasets under shared protocols, further enable the creation of cross-instrument models that are more resilient to variation. These developments support the vision of interoperable, globally harmonised phenotyping workflows, where data generated by different genebanks become directly comparable. Therefore, addressing calibration challenges becomes a pathway toward stronger international integration, better model reproducibility, and more reliable digital characterization of conserved germplasm.

Additional challenges arise from the need for long-term data curation, integration with existing databases, and the technical skills required to handle large, complex datasets, especially in organisations where informatics capacity is limited.

Despite all the listed challenges, the opportunities presented by seed phenomics are substantial. By enabling the rapid and precise assessment of a wide range of traits, phenomics allows genebanks to prioritise accessions with specific seed traits. Furthermore, the integration of phenomics with genomic and metabolomic data opens new avenues for trait discovery and functional characterisation, accelerating the development of crops with improved seed yield and nutritional quality of seed. These capabilities position phenomics as a cornerstone of modern genebank operations.

## 8 Future directions

The future of seed phenomics in genebanks lies in the integration of emerging technologies with scalable and cost-effective solutions that enhance the precision and efficiency of seed characterisation. One promising direction is the development of portable and automated phenotyping tools that can operate seamlessly. These tools, equipped with sensors like hyperspectral cameras, LiDAR, and thermal imagers, will enable the high-throughput assessment of diverse traits across large seed collections. By reducing reliance on fixed infrastructure, these innovations make advanced phenomics accessible to genebanks with limited resources, fostering broader adoption globally.

Another key area for growth is the advancement of data integration and artificial intelligence (AI) applications. The convergence of phenomics with multi-omics (genomics, metabolomics, and other)data will allow genebanks to build comprehensive datasets for complex trait analysis. AI and machine learning algorithms can further enhance this process by identifying patterns and associations within multidimensional datasets, accelerating trait discovery and the prioritisation of accessions. These capabilities are especially relevant for addressing pressing agricultural challenges, such as developing climate-resilient crops or improving nutritional quality. The creation of open-access phenomics databases and collaborative platforms will also facilitate global data sharing and cross-institutional research, enhancing the collective impact of genebanks.

The focus on standardization and community-driven protocols will be essential to ensuring the long-term success of seed phenomics. Such efforts will enable the harmonisation of phenomics practices across genebanks, ensuring data consistency and interoperability. Digital twins for seed collections (virtual, data-rich replicas of physical accessions) represent another transformative future direction for genebanks. By integrating real-time viability data, imaging-derived traits, environmental histories, and predictive models, digital twins would enable proactive management of regeneration needs, precise tracking of ageing dynamics, and more efficient utilization of genetic resources across global conservation networks. By embracing these future directions, seed phenomics will play a pivotal role in enhancing the utility and relevance of genebanks in the face of evolving global challenges.

The best recommendations for genebanks would be to prioritise cost-effective technologies like visible imaging, NIRS and thermal imaging for their routine high-throughput seed phenotyping. Genebank managers, however, should plan to adopt advanced imaging technologies such as HSI and X-ray imaging as their next priority and where possible combine technologies (e.g., hyperspectral and 3D imaging) to generate comprehensive datasets for both physical and chemical traits. An obvious step of preparation would be to invest in data management systems to store, analyze and share phenotyping data efficiently. Finally, they should engage in collaborations with phenomics research centers to access cutting-edge technologies and expertise such as machine learning and data science in order to enable the global network of genebanks adapt to the next wave of phenotyping and sensor technologies for knowledge sharing and advancement.

## 9 Concluding remarks

Seed phenomics has the potential to transform genebanks into dynamic hubs for genetic resource utilization. By adopting advanced technologies and fostering global collaborations and scalability, genebanks can enhance their role in ensuring food security and agricultural resilience in the face of climate change. This is an urgent need to modernize genebanks for more efficient seed and germplasm management.

## Data Availability

The original contributions presented in the study are included in the article/supplementary material. Further inquiries can be directed to the corresponding author.
